# How to make fluorescein strips

**Published:** 2018-11-09

**Authors:** Gilbert Arinda, Simon Arunga

**Affiliations:** 1 Ophthalmic Nurse, Mbarara University and Regional Referral Hospital Eye Centre, Mbarara, Uganda; 2 Clinical Lecturer and Ophthalmologist: Mbarara University and Regional Referral Hospital Eye Centre, Mbarara, Uganda.

Fluorescein strips are an essential diagnostic tool in eye care. They are useful for performing a number of procedures, such as measuring intraocular pressure, assessing dry eye and detecting corneal abrasions. Unfortunately, this basic item is not commonly available in many resource-limited settings. Here we describe how we make fluorescein strips at Mbarara University and Regional Referral Hospital Eye Centre.

## What you will need

- Sterile filter paper (e.g., Whatman no. 1)
- A sterile bowl, such as a kidney dish
- Fluorescein sodium powder (20 g)
- Distilled sterile water (100 ml)
- A pair of scissors
- Empty injectable vials or any other small, sealed containers
- A sterile surface
- Sterile gloves, mask and apron.

## Procedure

**Figure 1:**
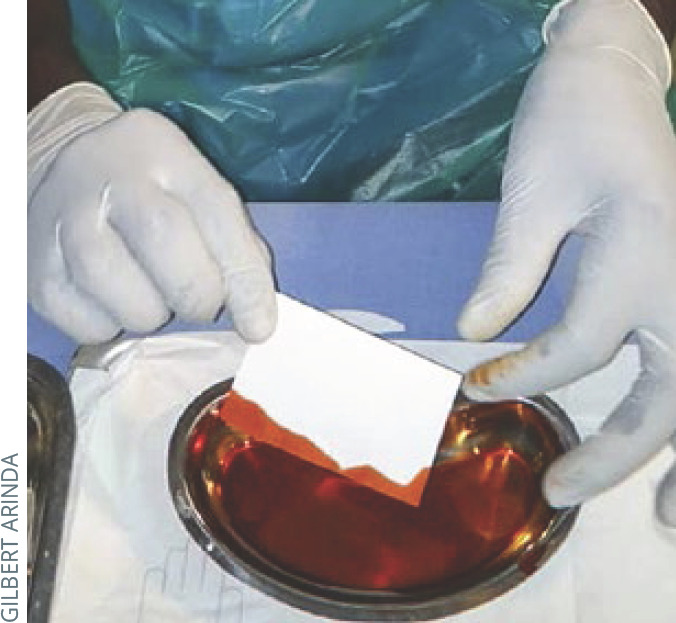
Dip the long edge of the filter paper rectangle in the fluorescein solution

**Figure 2:**
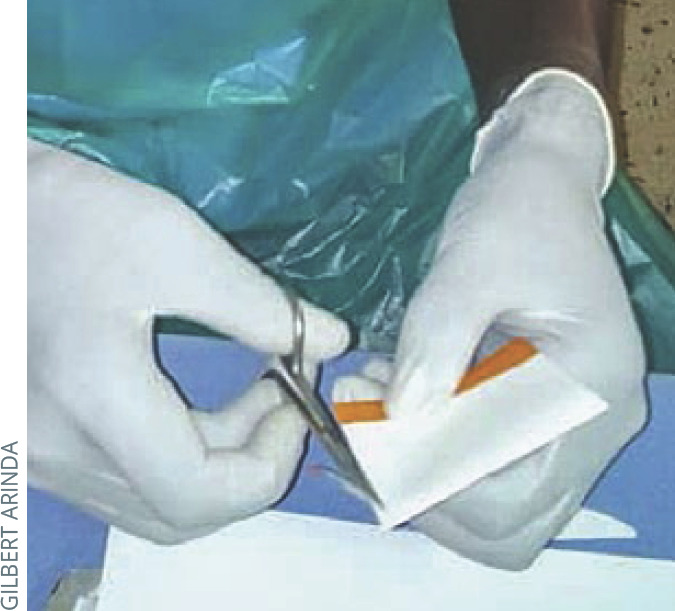
Cut the paper into strips with the dipped edge at one end

**Figure 3:**
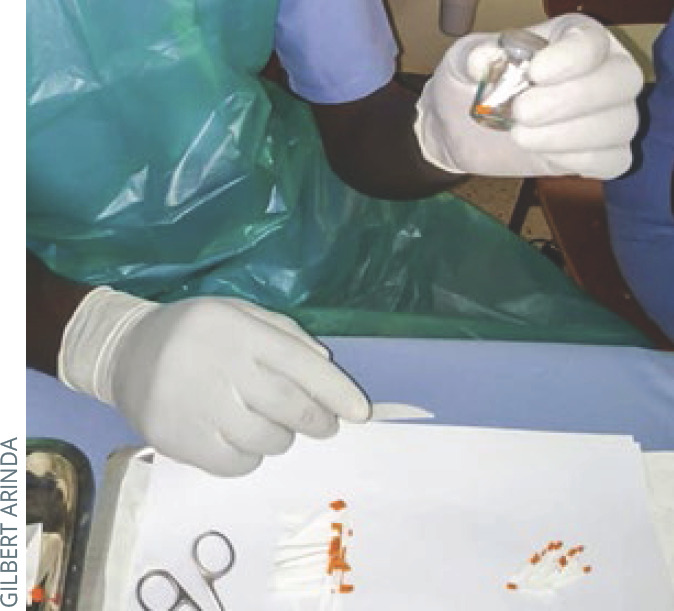
Store the strips in a sterile, sealed container

- Assemble all the materials on a clean tray
- Put on sterile gloves, mask and apron
- Prepare a 20% fluorescein solution by dissolving 20 g of fluorescein sodium powder in 100 ml distilled, sterile water
- Cut the filter paper into rectangles of approximately 5 cm wide and 8–10 cm long
- Pour a small amount of fluorescein solution into the bowl. Be careful not to spill, as fluorescein leaves stains
- Dip the long edge of the filter paper in the fluorescein solution and immediately remove it, as the solution spreads very quickly through the paper (Figure 1)
- Place the dipped filter papers onto a sterile surface to dry
- Once they are dry, use a pair of scissors to cut the paper into strips, with the dipped edge at one end (Figure 2)
- Store the strips in a sterile, sealed container (Figure 3).

